# PGRN is involved in macrophage M2 polarization regulation through TNFR2 in periodontitis

**DOI:** 10.1186/s12967-024-05214-7

**Published:** 2024-04-30

**Authors:** Liguo Zhang, Fujiao Nie, Jingjing Zhao, Shutong Li, Wenchuan Liu, Hongmei Guo, Pishan Yang

**Affiliations:** 1 Department of Periodontology, School and Hospital of Stomatology, Cheeloo College of Medicine, Shandong Key Laboratory of Oral Tissue Regeneration & Shandong Engineering Laboratory for Dental Materials and Oral Tissue Regeneration & Shandong Provincial Clinical Research Center for Oral Diseases, Shandong University, Jinan, Shandong 250012 China; 2 Section of Infection and Immunity, Herman Ostrow School of Dentistry, University of Southern California, Los Angeles, CA USA

**Keywords:** PGRN, Macrophage polarization, TNFR2, Periodontitis, Multiple fluorescence immunohistochemistry

## Abstract

**Background and objective:**

Progranulin (PGRN), a multifunctional growth factor, plays indispensable roles in the regulation of cancer, inflammation, metabolic diseases, and neurodegenerative diseases. Nevertheless, its immune regulatory role in periodontitis is insufficiently understood. This study attempts to explore the regulatory effects of PGRN on macrophage polarization in periodontitis microenvironment.

**Methods:**

Immunohistochemical (IHC) and multiplex immunohistochemical (mIHC) stainings were performed to evaluate the expression of macrophage-related markers and PGRN in gingival samples from periodontally healthy subjects and periodontitis subjects. RAW264.7 cells and bone marrow-derived macrophages (BMDMs) were polarized towards M1 or M2 macrophages by the addition of LPS or IL-4, respectively, and were treated with or without PGRN. Real-time fluorescence quantitative PCR (qRT-PCR), immunofluorescence staining (IF), enzyme-linked immunosorbent assay (ELISA), and flow cytometry were used to determine the expressions of M1 and M2 macrophage-related markers. Co-immunoprecipitation was performed to detect the interaction between PGRN and tumor necrosis factor receptor 2 (TNFR2). Neutralizing antibody was used to block TNFR2 to confirm the role of TNFR2 in PGRN-mediated macrophage polarization.

**Results:**

The IHC and mIHC staining of human gingival slices showed a significant accumulation of macrophages in the microenvironment of periodontitis, with increased expressions of both M1 and M2 macrophage markers. Meanwhile, PGRN was widely expressed in the gingival tissue of periodontitis and co-expressed mainly with M2 macrophages. In vitro experiments showed that in RAW264.7 cells and BMDMs, M1 markers (CD86, TNF-α, iNOS, and IL-6) substantially decreased and M2 markers (CD206, IL-10, and Arg-1) significantly increased when PGRN was applied to LPS-stimulated macrophages relatively to LPS stimulation alone. Besides, PGRN synergistically promoted IL-4-induced M2 markers expression, such as CD206, IL-10, and Arg1. In addition, the co-immunoprecipitation result showed the direct interaction of PGRN with TNFR2. mIHC staining further revealed the co-localization of PGRN and TNFR2 on M2 macrophages (CD206+). Blocking TNFR2 inhibited the regulation role of PGRN on macrophage M2 polarization.

**Conclusions:**

In summary, PGRN promotes macrophage M2 polarization through binding to TNFR2 in both pro- and anti-inflammatory periodontal microenvironments.

**Supplementary Information:**

The online version contains supplementary material available at 10.1186/s12967-024-05214-7.

## Introduction

Periodontitis is one kind of chronic inflammatory disease with high incidence in adults, characterized by inflammation of gingival tissue and destruction of alveolar bone [1–6]. The destruction of periodontal tissues consequently results in tooth loosening, even falling off, which dreadfully impairs oral masticatory functions, such as chewing and pronunciation [7, 8]. The pathogenesis of periodontitis is rather intricate and has not been entirely explained. Increasing pieces of evidence indicate that a predominant amount of macrophages infiltrate in the gingival tissue of periodontitis and the polarization state of macrophages plays a distinct role in the progression of periodontitis [9–11]. In detail, M1 macrophages mainly take part in the destructive process of periodontitis by evoking secretion of diverse pro-inflammatory factors, such as interleukin-6 (IL-6), tumor necrosis factor-α (TNF-α) and inducible nitric oxide synthase (iNOS). These products all together bring out the aggravation of inflammation and absorption of alveolar bone [12, 13]. To the delight, the transformation of macrophages from M1 towards M2 can alleviate the inflammation state attributed to the production of transforming growth factor-β (TGF-β) and interleukin-10 (IL-10) [14, 15]. Hence, appropriate regulation of macrophage polarization, that is, inhibiting M1 and promoting M2, may become a promising strategy for the treatment of periodontitis.

Progranulin, a key protein in the regulation of inflammatory responses, participates in the regulation of inflammatory response, tissue damage, and other pathophysiological progress [16–21]. Recently, the anti-inflammation and bone regeneration promotion effects of PGRN in periodontitis have been studied by our research group. The previous studies indicate that patients with periodontitis show higher expression of PGRN in gingival crevicular fluid and gingival tissue. Besides, local administration of PGRN relieves periodontal tissue inflammation and reduces the loss of alveolar bone in rat periodontitis [4]. Also, the utilization of exogenous PGRN in periodontal bone defect models in rats and dogs can diminish the infiltration of inflammatory cells, regulate immune reaction, and enhance alveolar bone regeneration [3, 22]. The in-vitro studies have shown that PGRN can reverse LPS-induced polarization of RAW264.7 cells towards M1 [18]. All these studies suggest that PGRN exerts anti-inflammation, immunoregulation, and periodontal bone regeneration effects in periodontitis. However, the regulatory effect of PGRN on macrophage M2 polarization in the periodontitis microenvironment remains largely unclear. In addition, PGRN has been reported to have several binding sites, including TNFRs, sortilin, et al., but the potential binding receptor of PGRN in the periodontitis microenvironment needs to be clarified [23]. In this study, we first studied the expressions and co-localization of PGRN and macrophage M1 and M2 markers in healthy and periodontitis gingiva to analyze the possibility of PGRN in modulating macrophage polarization, further verified our hypothesis through cell experiments, and finally identify the binding site of PGRN in the periodontitis microenvironment.

## Results

### The enhanced macrophage infiltration and PGRN expression in periodontitis

**Fig. 1:**
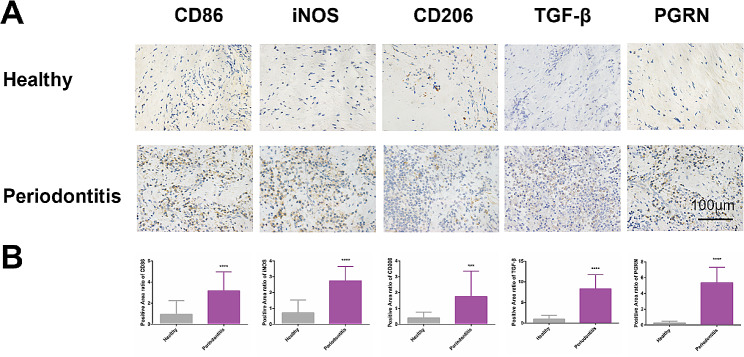
Expressions of PGRN and macrophage markers in gingival tissue. **(A)**: Expression of CD86, iNOS, CD206, TGF-β, and PGRN in gingival tissue, healthy and periodontitis gingiva groups. **(B)**: Semi-quantitative analysis of the positive expression area in the IHC stainings, *n* = 20. ***: *P* < 0.001, ****: *P* < 0.0001

We first detected the expression of PGRN and macrophage-related markers in the gingiva of healthy and periodontitis subjects. The IHC staining showed that the expressions of PGRN, M1 macrophage-associated markers (CD86 and iNOS), and M2 macrophage-associated markers (TGF-β and CD206) were significantly higher in periodontitis than that in healthy gingiva **(**Fig. 1A**)**. The semi-quantitative analysis results further showed that compared with healthy gingiva, the expression of iNOS-positive areas in periodontitis group increased to 3.79 times, CD86 increased to 3.36 times, TGF-β increased to 8.37 times, CD206 increased to 4.31 times, and PGRN increased to 19.43 times (Fig. 1B). These results prompted a distinct expression profile (macrophage markers and PGRN) between healthy and periodontitis gingiva.

### PGRN co-expresses with macrophages in periodontitis

**Fig. 2:**
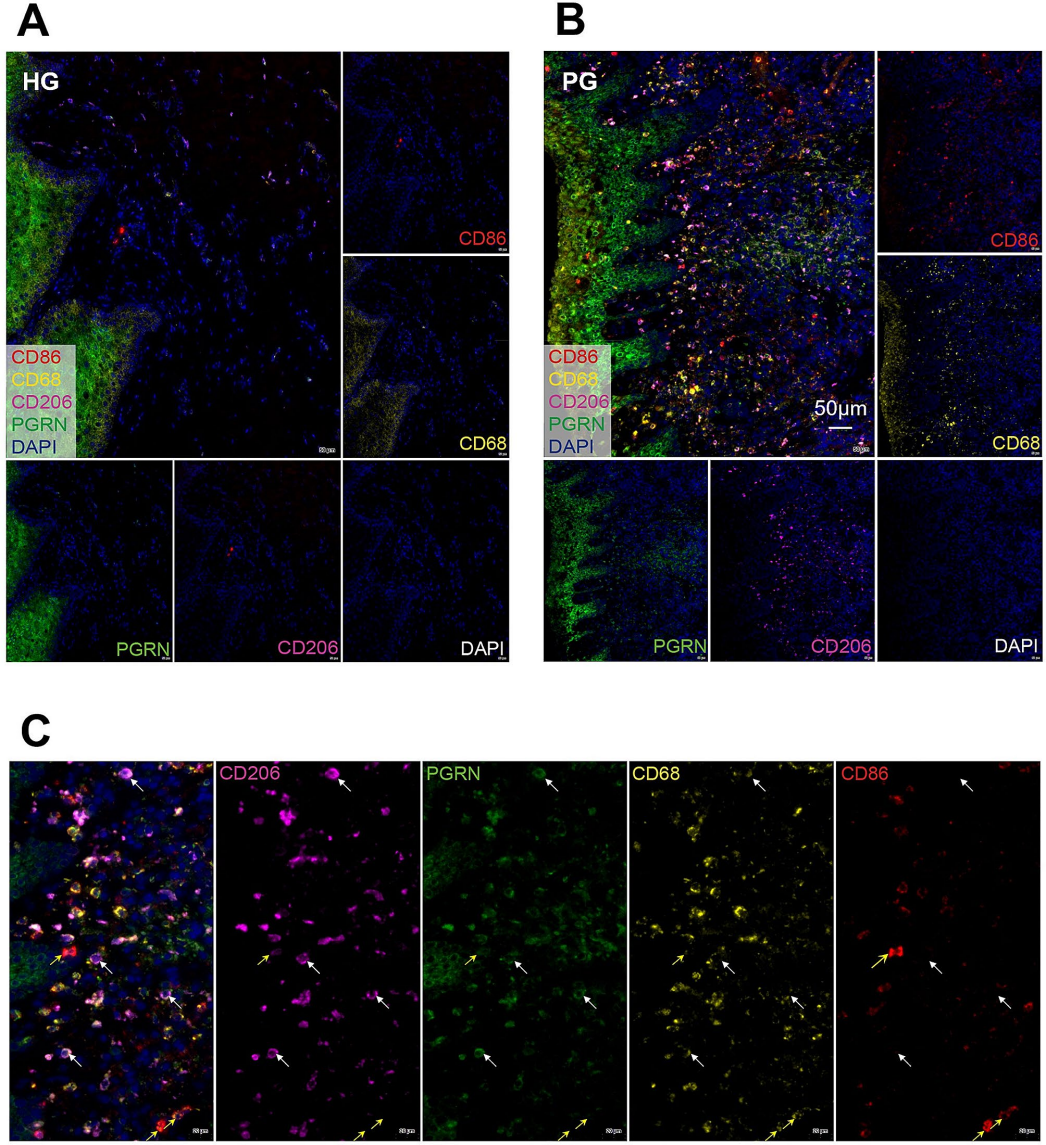
Co-expression of PGRN and macrophage markers in gingival tissue. **(A) (B)**: Co-expression of PGRN, CD68, CD86, and CD206 in healthy gingiva (HG) and periodontitis gingiva (PG), respectively. **(C)**: Higher magnification image of gingiva shown in the upper panel, PG. White arrow: M2 type macrophage (CD68 + CD206+). Yellow arrow: M1 type macrophage (CD68 + CD86+)

To analyze the possibility of PGRN in regulating macrophage polarization, mIHC was performed to evaluate the co-expression of PGRN and macrophage markers in gingiva. As shown in Fig. 2, both M1 (CD68 + CD86+) and M2 (CD68 + CD206+) macrophages were increased in gingiva with periodontitis compared to healthy gingiva (Fig. 2A and B), which confirmed the results of IHC. More importantly, PGRN was poorly expressed in M1 macrophages (CD68 + CD86+), but strongly expressed in M2 macrophages (CD68 + CD206+) (Fig. 2C). This implies that PGRN may participate in macrophage polarization regulation.

### PGRN inhibits LPS-stimulated macrophage polarization towards M1 and promotes it toward the M2

**Fig. 3:**
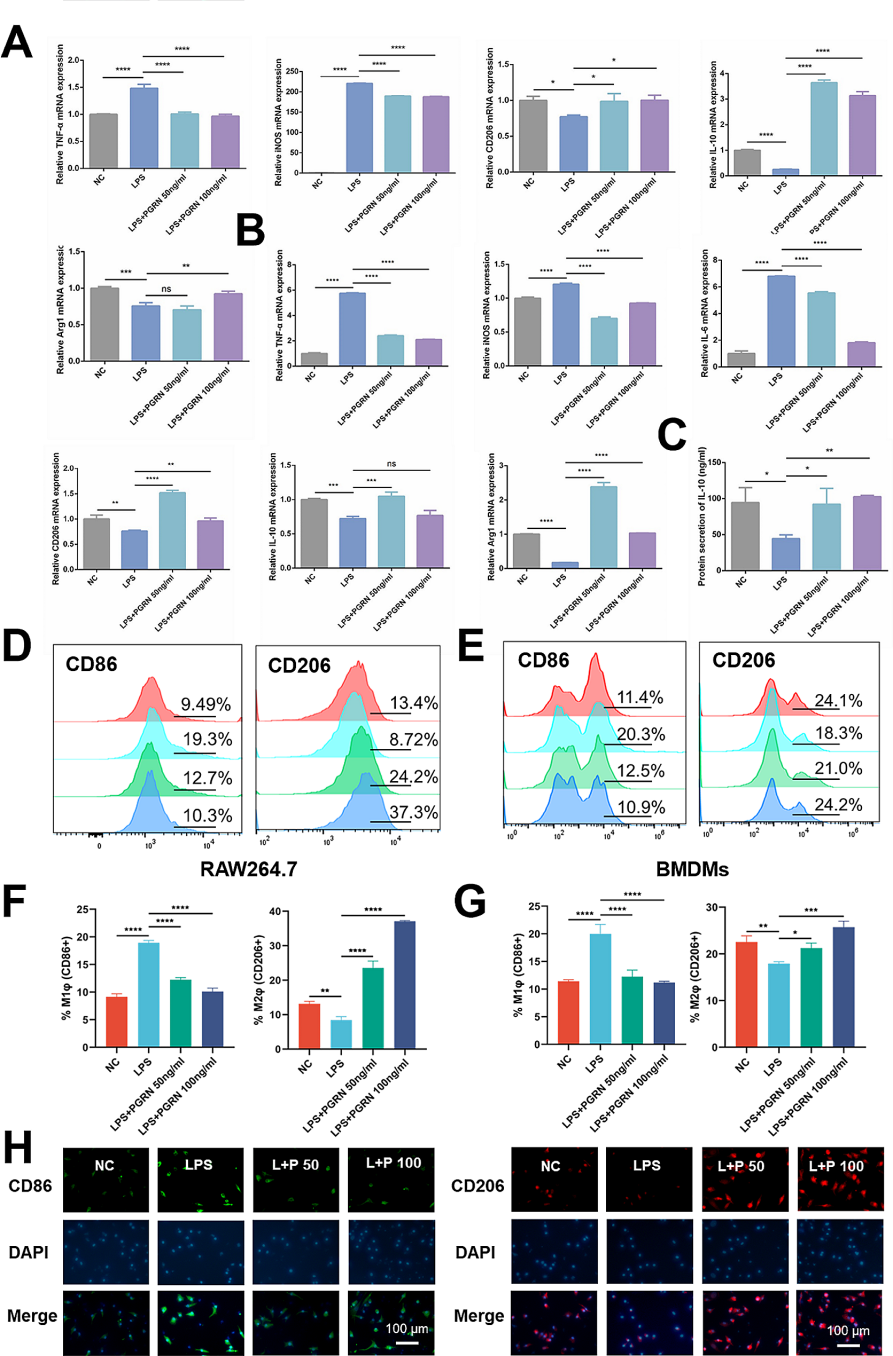
The regulation of PGRN on LPS-stimulated macrophage polarization. **(A)**: The mRNA expressions of TNF-α, iNOS, CD206, IL-10, and Arg-1 in RAW264.7 cells. **(B)**: The mRNA expressions of TNF-α, iNOS, IL-6, CD206, IL-10, and Arg-1 in BMDMs. **(C)**: The IL-10 protein release measured by ELISA in supernatant samples, RAW264.7 cells. **(D)(F)**: Expressions and quantitative analysis of CD86 and CD206 markers in RAW264.7 assessed by flow cytometry. From top to bottom, NC, LPS, LPS + PGRN 50 ng/mL, and LPS + PGRN 100 ng/mL respectively. **(E)(G)**: Expressions and quantitative analysis of CD86 and CD206 markers in BMDMs assessed by flow cytometry. From top to bottom, NC, LPS, LPS + PGRN 50 ng/mL, and LPS + PGRN 100 ng/mL respectively. **(H)**: Immunofluorescence staining of CD86 and CD206 in BMDMs. L + P 50: LPS + PGRN 50 ng/mL, L + P 100: LPS + PGRN 100 ng/mL. * *P* < 0.05, ** *P* < 0.01, *** *P* < 0.001, **** *P* < 0.0001, and ns: no significance

To confirm the regulatory effect of PGRN on macrophage polarization in vitro, both RAW264.7 and BMDMs were pre-stimulated with LPS and then treated with or without exogenous PGRN. The concentrations of exogenous PGRN, 50 ng/mL and 100 ng/mL, were set according to our previous studies [18]. In addition, before using BMDMs, we conducted macrophage identification on the extracted BMDMs. The result of flow cytometry showed that there were high expressions of CD11b and F4/80 (Fig.S1, in supporting information), indicating that the BMDMs we used met the criteria for identification. qRT-PCR results showed that enhanced mRNA expression of M1 macrophage-associated TNF-α, iNOS, and IL-6 induced by LPS was reduced after the treatment of PGRN (*P* < 0.05). Also, PGRN reversed LPS-inhibited mRNA expression of CD206, IL-10, and Arg-1, which were associated with M2 macrophages (*P* < 0.05) (Fig. 3A and B). The ELISA result showed a similar trend that PGRN could significantly rescue LPS-suppressed secretion of IL-10 in RAW264.7 cells (*P* < 0.05) (Fig. 3C). Flow cytometry demonstrated a sharp decrease in the number of CD86 + cells (in both RAW264.7 and BMDMs) after treatment with PGRN, even dropping by about half in the 100 ng/mL PGRN group compared to the LPS stimulation alone (Fig. 3D and F). At the same time, PGRN was able to reverse the downregulation of CD206 + cells stimulated by LPS, increasing the proportion of CD206 + macrophages from 8.72% to 37.3% (100 ng/mL PGRN) in RAW264.7 and the proportion of CD206 + macrophages from 18.3 to 24.2% (100 ng/mL PGRN) in BMDMs (Fig. 3E and G). Furthermore, immunofluorescence staining of BMDM cells showed that PGRN reduced the expression of CD86 + cells and promoted polarization toward the M2 phenotype (CD206 + cells) (Fig. 3H). These results demonstrate that PGRN not only inhibits LPS-stimulated macrophage M1 polarization but also reverses LPS-suppressed macrophage M2 polarization.

### PGRN synergistically facilitates IL-4-induced M2 polarization

**Fig. 4:**
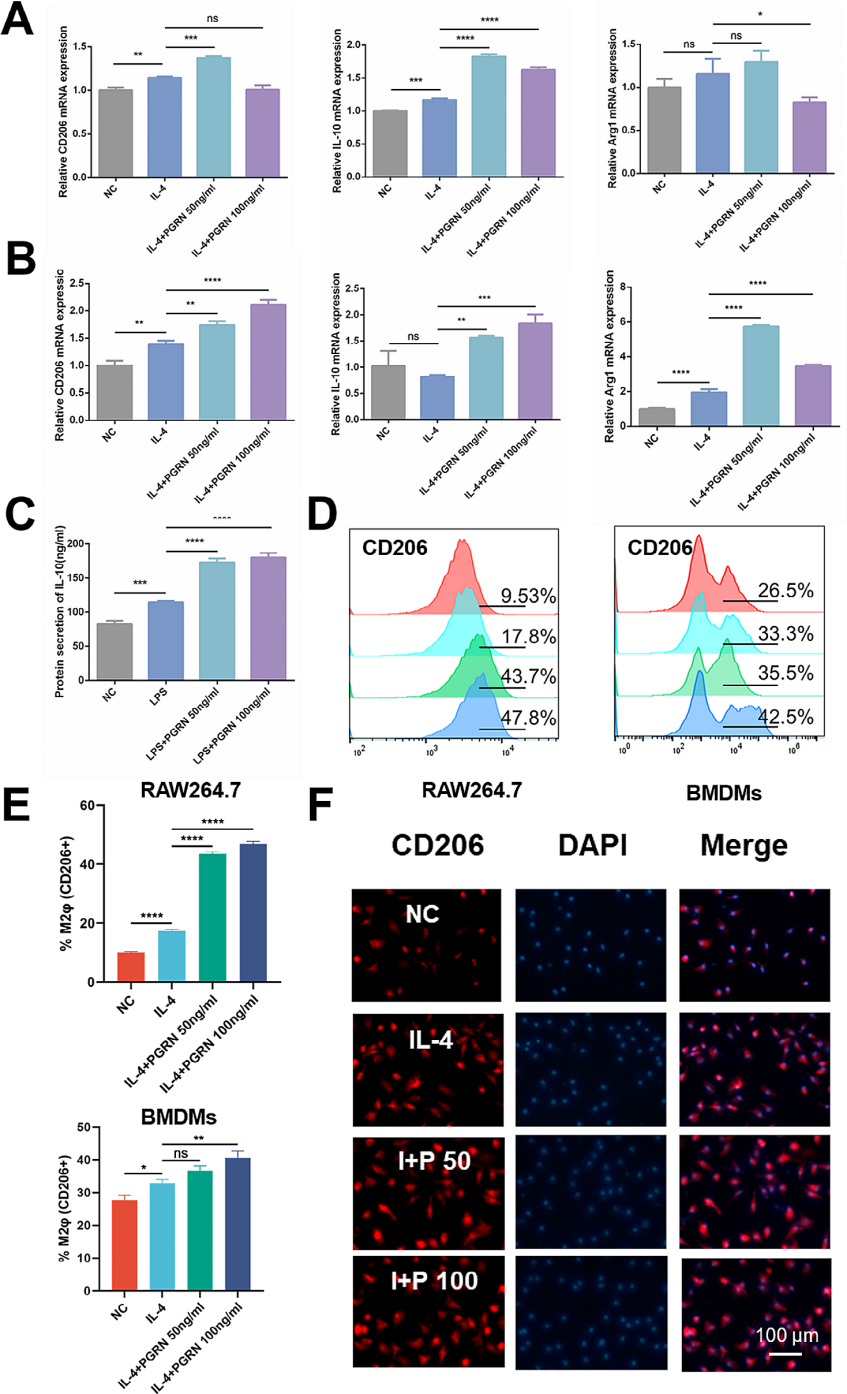
The regulation of PGRN on IL-4-stimulated macrophage polarization. **(A)**: The mRNA expressions of CD206, IL-10, and Arg-1 in RAW264.7 cells. **(B)**: The mRNA expressions of CD206, IL-10, and Arg-1 in BMDMS. **(C)**: The IL-10 protein release measured by ELISA in supernatant samples, RAW264.7 cells. **(D)(E)**: Expressions and quantitative analysis of CD206 marker in RAW264.7 (left) and BMDMs (right) assessed by flow cytometry. From top to bottom, NC, IL-4, IL-4 + PGRN 50 ng/mL, and IL-4 + PGRN 100 ng/mL respectively. **(F)**: Immunofluorescence staining of CD206 in BMDMs. I + P 50: IL-4 + PGRN 50 ng/mL, I + P 100: IL-4 + PGRN 100 ng/mL. * *P* < 0.05, ** *P* < 0.01, *** *P* < 0.001, **** *P* < 0.0001, and ns: no significance

To analyze the effect of PGRN on macrophage M2 polarization in the anti-inflammatory (IL-4-induced) microenvironment, RAW264.7 cells, and BMDMs were pretreated with IL-4 with or without PGRN, and the expressions of M2 polarization markers were subsequently detected. qRT-PCR results showed that the addition of PGRN boosted the mRNA expressions of M2 macrophage-associated markers, including CD206, IL-10, and Arg-1 (*P* < 0.05) (Fig. 4A and B). ELISA results also conveyed that the addition of PGRN at 50 ng/mL and 100 ng/mL promoted IL-10 secretion compared to the IL-4 group (*P* < 0.05) (Fig. 4C). Flow cytometry results showed that the addition of PGRN could significantly promote the expression of CD206 in both RAW264.7 and BMDM cells, compared with the IL-4 stimulated group. In the RAW264.7 cell line, PGRN increased the proportion of CD206 + macrophages over twice with the addition of 100 ng/mL PGRN. In BMDMs, PGRN increased the proportion of CD206 + macrophages from 33.3 to 42.5% (Fig. 4D and E). Similarly, immunofluorescence staining results showed that the number of CD206 + cells was remarkably increased in anti-inflammatory (IL-4-induced) microenvironment after the addition of PGRN (Fig. 4F). All these results indicate that PGRN could synergistically enhance the effect of IL-4 on promoting macrophage polarization towards M2.

### TNFR2 mediates the effect of PGRN on M2 macrophage polarization

**Fig. 5:**
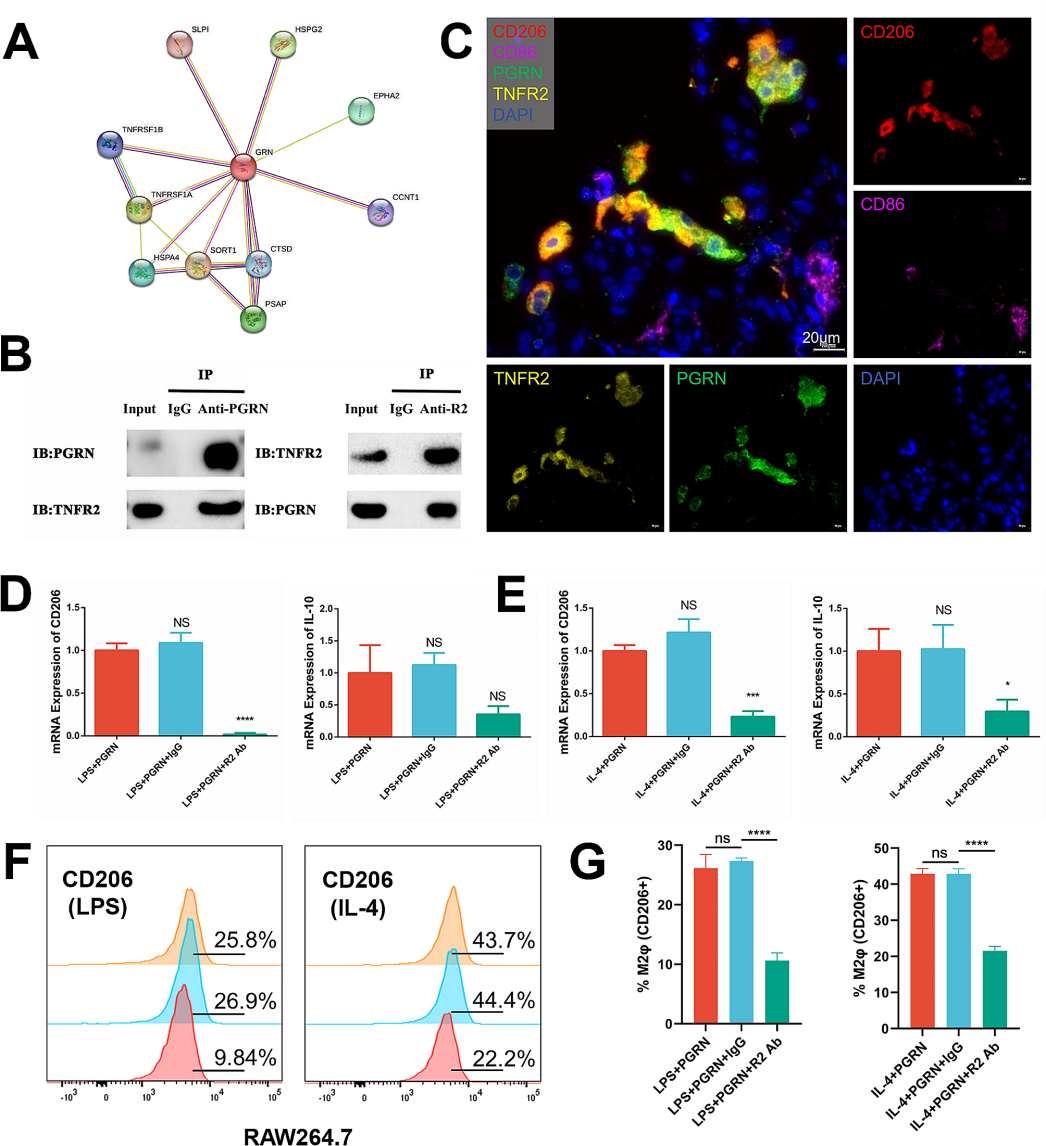
TNFR2 mediates the effect of PGRN on M2 macrophage polarization **(A)**: Protein-protein interaction (PPI) network of PGRN. Network nodes represent proteins. Edges represent protein-protein associations **(B)**: The co-immunoprecipitation result of PGRN and TNFR2 interaction. **(C)**: mIHC staining for the co-localization of PGRN and TNFR2 on CD68 + macrophages in periodontitis gingiva. **(D)**: The mRNA expression of CD206 and IL-10 in RAW264.7 after pre-treatment with TNFR2 neutralizing antibody and addition of LPS + PGRN. **(E)**: The mRNA expression of CD206 and IL-10 in RAW264.7 after pre-treatment with TNFR2 neutralizing antibody and addition of IL-4 + PGRN. **(F)(G)**: Expressions and quantitative analysis of CD206 marker in RAW264.7 assessed by flow cytometry. LPS + PGRN groups (left) and IL-4 + PGRN groups (right). From top to bottom, LPS + PGRN, LPS + PGRN + IgG and LPS + PGRN + R2-Ab & IL-4 + PGRN, IL-4 + PGRN + IgG and IL-4 + PGRN + R2-Ab, respectively. * *P* < 0.05, *** *P* < 0.001, **** *P* < 0.0001, and ns: no significance

The above results show that PGRN can regulate macrophage polarization in periodontitis microenvironment. However, the subsequent regulatory mechanisms are not yet clear, especially regarding the binding sites of PGRN on macrophages in the periodontitis microenvironment. In this part, we first networked all potential binding sites of PGRN by using protein-protein interaction (PPI) analysis and the result exhibited several potential binding sites for PGRN, including TNFRs, sortilin, SLP1, and EPHA2 et al. **(**Fig. 5A**)**. However, these binding sites represent obvious tissue-specific and disease-specific characteristics. For example, SLP1, sortilin, and EPHA2 are specifically distributed in the nervous system and function in neurological diseases [24–26]; CCNT1 is related to HIV infections [27]; while TNFRs (TNFR1, TNFR2) are associated with inflammatory regulation, of which TNFR2 usually involves anti-inflammatory responses [28, 29]. Therefore, we considered TNFR2 as a candidate for mediating PGRN-promoted M2 macrophage polarization.

To verify that PGRN can directly bind to the TNFR2 on macrophages, we conducted co-immunoprecipitation experiments. The results showed that PGRN and TNFR2 were detected in the input groups, indicating the presence of both proteins in the samples. The IgG groups did not show any bands, ruling out the possibility of non-specific binding. However, the anti-PGRN group and the anti-TNFR2 group showed significant bands at the positions, indicating the existence of an interaction between PGRN and TNFR2 **(**Fig. 5B**)**. mIHC was further used to confirm the binding of PGRN and TNFR2 in the periodontitis microenvironment. The results showed that PGRN bound to TNFR2 on the surface of both phenotypes of macrophages, including M1 and M2, but this binding was more pronounced in M2 macrophages **(**Fig. 5C**)**. This may be related to the higher expression of TNFR2 on M2 macrophages, which has been confirmed by in-vitro experiments, that is, during the induction of macrophages into M1, the mRNA expression of TNFR2 decreased, while it significantly increased during the induction into M2 (Fig. S2, in supporting information).

After verifying the binding of PGRN and TNFR2 in the periodontal microenvironment, we pre-treated RAW264.7 macrophage with TNFR2 neutralizing antibodies to block the binding and to observe whether PGRN-TNFR2 binding mediated the pro-M2 polarization effects. qRT-PCR results showed that after pretreatment with TNFR2 neutralizing antibody, the mRNA expression of M2-related markers, CD206 and IL-10 significantly decreased in both pro-inflammatory and anti-inflammatory microenvironment (*P* < 0.05) **(**Fig. 5D and E). The results of flow cytometry also show a similar trend. In both pro-inflammatory and anti-inflammatory microenvironments, the use of TNFR2-neutralizing antibodies led to a decrease in the proportion of the CD206 + macrophages in RAW264.7. **(**Fig. 5F and G). The results all together reveal that TNFR2 mediates the role of PGRN in promoting the polarization of M2 macrophages in the periodontitis microenvironment.

## Discussion

Given the pro-inflammatory action of M1 macrophages and the anti-inflammatory effect of M2 macrophages, regulating the transformation of macrophage polarization may become a promising strategy for the treatment of periodontitis. Zhou et al. found that the M1/M2 ratio in the gingival tissues of patients with chronic periodontitis significantly increased and clinical parameters, such as periodontal pocket depth, were associated with the ratio [13]. Naqvi et al. identified that lower expression of M1 markers (TNF-α, STAT1) was observed after periodontal treatment [30]. These studies present a dynamic shift between the macrophage polarization and periodontitis processes. However, the regulatory mechanisms of the shift remain largely unclear. The present study reveals that PGRN promotes M2 polarization and inhibits M1 polarization of macrophage in periodontitis microenvironment by binding to TNFR2, further strengthening the therapeutic potentials of PGRN for periodontitis.

In detail, based on the IHC and mIHC stainings of human gingival samples, we proposed the hypothesis: the expression of PGRN may be related to macrophage polarization (positive in expressions; co-expression of PGRN and macrophage markers). To validate the hypothesis, we then conducted in-vitro experiments, and the results confirmed that PGRN regulated macrophage polarization towards the M2 phenotype, providing a theoretical basis for the use of PGRN in the treatment of periodontitis. Considering that PGRN has a few downstream binding sites that present with tissue specificity, further clarifying the downstream binding sites of PGRN in the periodontal microenvironment is of great significance because different binding sites may cause different cascade reactions. Co-immunoprecipitation, mIHC staining, and TNFR2 neutralizing antibody blocking, all together revealed that TNFR2 mediated the role of PGRN in promoting the polarization of M2 macrophages in the periodontitis microenvironment. And we infer that the subsequent cascade of events may be as follows. The first step is the binding of PGRN to the specific domain of TNFR2. Jian et al. explained that PGRN could interact with the cysteine-rich domain 2 (CRD2) and cysteine-rich domain 3 (CRD3) in TNFR2, and this kind of biological effect was exerted only if the protein structure was folded [31]. After the binding, the formation of TNF-alpha/TNFR complexes and the TNF-alpha signaling are disrupted, thereby preventing the activation of downstream signaling pathways, such as the ubiquitin-proteasome pathway involved in NF-κB activation, which leads to its inhibition and subsequent attenuation of inflammatory responses [32]. PGRN can also modulate the activity of MAPKs, including ERK, JNK, and p38 MAPK [33, 34]. By inhibiting the phosphorylation of these kinases, PGRN can attenuate the downstream signaling events that lead to the production of anti-inflammatory cytokines and chemokines. In addition, PGRN binding to TNFRs may also trigger intracellular signaling events that promote the activation of anti-inflammatory pathways, such as the PI3K-Akt pathway [35], leading to M2 polarization and suppression of inflammation.

Collectively, this study primarily reveals that PGRN is involved in regulating the transformation of macrophage towards M2 in periodontitis and TNFR2 mediates, at least in part, this process. This may be one of the important mechanisms by which PGRN exerts its anti-inflammatory, immune-regulatory, and periodontal regeneration effects.

However, some limits exist in this study. (1) Our research results show that PGRN is derived from both epithelial cells and macrophages. Whether PGRN from different sources exerts different biological effects is largely unknown. We speculate that PGRN may exert its effects predominantly through autocrine signaling, with epithelial cell-derived PGRN involved in maintaining epithelial cell homeostasis, and macrophage-derived PGRN involved in macrophage polarization. Evidences are needed for further validation. (2) Modulating Th17/Treg imbalance is becoming a hot topic in the periodontal study field. In another parallel experiment, our research group established a beagle periodontitis model and confirmed that PGRN can also modulate CD4 + T differentiation into Treg [22], while in-vitro evidence needs to be provided in the future. (3) Macrophages in the body exhibit significant heterogeneity, usually displaying tissue-specific phenotypes. Single-cell sequencing of gingival tissues from periodontitis patients revealed three types of macrophages, including PRDM1 + Macro, NLRP3 + Macro, and C1QA + Macro [36]. Another study showed that CD301b + macrophages have specificity in the bone immunological microenvironment and play a crucial role in periodontal bone remodeling [37]. How PGRN modulates the new macrophage subpopulations in periodontitis waits for further investigation. (4) Based on our studies, PGRN shows promising prospects for periodontitis therapy. Also, other evidence confirmed that PGRN could be a promising biomarker candidate for periodontal disease [38]. However, the optimal usage scenarios and safety of the PGRN need to be elucidated.

## Methods and materials

### Clinical sample collection and inclusion criteria

The gingival tissues were obtained during crown lengthening surgery (healthy gingiva) and gingivectomy (gingiva with periodontitis) in the Hospital of Stomatology, Shandong University from December 2022 to January 2023. After strict disinfection and anesthesia preoperatively, gingiva without clinical value but suitable for research were excised. The research was approved by the Ethics Committee of the Hospital of Stomatology, Shandong University (NO: 20220318). All subjects signed the informed consent form before participating in the study.

The gingiva of the periodontitis group (*n* = 20) was taken from the patients with periodontitis (Stage III, Grade C) that had not been controlled after basic treatment. The gingiva of the control group (*n* = 20) came from periodontally healthy volunteers with periodontal probing depth ≤ 3 mm and no gingiva inflammation during the crown extension surgery. The ages of volunteers ranged from 18 to 65 years old.

Exclusion criteria: diabetes, abnormal coagulation mechanism, abnormal immune function, or other systemic diseases that affect the progress and prognosis of periodontal disease; Smokers or quit smoking for less than 6 months; Pregnant and lactating women; Take NSAIDs or antibiotics within 3 months before the first diagnosis.

### IHC staining

The tissue samples were fixed with 4% paraformaldehyde solution (Biosharp, Beijing, China) for 24∼48 h, then were sliced with a thickness of 4 μm. IHC staining of CD86, iNOS, TGF-β, CD206, and PGRN was performed following the manufacture of an immunohistochemical kit (Zhongshan Jinqiao Biotechnology, Beijing, China). The antibodies were listed in Table 1 (in Supporting Information). ImageJ was used to analyze IHC images, and the IHC Toolbox plugin was applied to standardize the selection of positive staining and eliminate any subjective factors. Finally, the percentage of the positive area was determined as the indicator of PGRN, iNOS, CD86, TGF-β, and CD206 positive expression. GraphPad Prism 8.0 was used to analyze the differences in the expression of various target molecules between the healthy group and the periodontitis group.

### mIHC staining

mIHC was performed using an Opal 7-plex fIHC kit (PerkinElmer, Waltham, MA, USA)). Human gingival tissue Sect. (4 μm) from healthy individuals and patients with periodontitis were labeled with primary antibodies against CD68, CD86 CD206, PGRN, and TNFR2, followed by secondary antibodies. The antibodies were listed in Table 1 (in Supporting Information). Subsequently, the fluorophore-conjugated tyramide amplification system (PerkinElmer) was used for signal amplification, and DAPI was used to counterstain the nuclei. Slides were scanned using the TissueFAXS imaging system (TissueGnostics GmbH, Vienna, Austria).

### Cell culture

The RAW264.7 murine macrophage cell line was purchased from the National Collection of Authenticated Cell Cultures (Shanghai, China), and was cultured in DMEM medium (hyclone, Logan, UT, USA) containing 10% fetal bovine serum (FBS, BioInd, Kibbutz, Israel) [18]. Primary bone marrow-derived macrophages (BMDM) were isolated from the bone marrow of C57Bl/6 mice tibia and femur, and then cultured in the DMEM medium supplemented with 20% FBS and M-CSF (50 ng/mL) for 7 days to differentiate into BMDMs [18]. The incubation condition of the incubator is set as 5% CO_2_ concentration and 37 ℃ temperature. BMDM was identified by flow cytometry before use, with the identification markers being CD11b and F4/80.

RAW264.7 cells and BMDMs were inoculated into six-well plates at a density of 2х10^5^ cells per well. 24 h after planking, the cells were treated according to different grouping methods: control, LPS-induced (to induce M1 macrophage, 100 ng/mL), LPS + 50 ng/mL PGRN, LPS + 100 ng/mL PGRN; and control, IL-4-induced (to induce M2 macrophage, 20 ng/mL), IL-4 + 50 ng/mL PGRN, IL-4 + 100 ng/mL PGRN. To explore the role of TNFR2 signaling, TNFR2 monoclonal antibody was applied for 24 h before the PGRN application at a concentration of 1 µg/mL. The control group was given IgG addition to exclude other possible influences. After 48 h of stimulation, the cells were collected for further use.

### Flow cytometry

The cells were adjusted to the number of 1 × 10^6^ per sample. Use CD16/32 antibody (Biolegend, San Diego, CA, USA) to block nonspecific F_C_-mediated interaction. Next, cells were incubated with CD86 antibody (Biolegend, San Diego, CA, USA) on ice for 30 min in the dark. Then cells were fixed, permeabilized, and incubated with CD206 antibody (Biolegend, San Diego, CA, USA) at room temperature for 30 min. Finally, use 500 µl PBS buffer containing 3% FBS to resuspend the cells before it being analyzed on flow cytometry (BD Biosciences, San Diego, CA, USA) [18].

### qRT-PCR

Total RNA was extracted with Trizol (Takara, Kusatsu, Japan); mRNA was reverse-transcribed to cDNA using the PrimeScript™ RT reagent Kit with gDNA Eraser (Yeasen, China). the qRT-PCR reaction was performed with SYBR Mix (Yeasen, China) on a Light Cycler Roche 480 II Real-Time PCR System (Roche, Basel, Switzerland) in triplicate and the annealing temperature was set at 60 ℃. The primer sequences were listed in Table 2 (in Supporting Information). GAPDH was used as the internal control. Data were analyzed using the 2^−(ΔΔCt)^ method [18].

### ELISA

The cell supernatant and standards were added to the wells of micro-ELISA plates and incubated with the specific detection antibody for 1.5 h. Then the microplates were washed to remove unbound antibodies, followed by incubation with an HRP-conjugated secondary antibody for an additional hour. Subsequently, the plates were treated with a chromogenic substrate and analyzed using a microplate reader, with absorbance readings recorded at 450 nm.

### Protein-protein interaction (PPI) network analysis

To examine PGRN’s binding receptor, functional enrichment analysis of the protein-protein interaction network was performed using STRING online datasets (https://cn.string-db.org/). The species selected is human, and the target gene/protein is progranulin.

### Co-immunoprecipitation (Co-IP)

Cells were collected and lysed in an appropriate precooled IP buffer. The cell lysate was centrifuged after being frozen for 30 min. Took 50 µl lysate supernatant as input. The rest was incubated with the indicated antibody against PGRN (Abcam, USA), TNFR2 (proteintech, USA), or IgG negative control (Santa, USA) on a rotating device at 4 ℃ for 2 h. Protein A/G Plus Agarose Beads (Santa, sc-2003) were washed 2 times with IP buffer and added to the cell lysate-antibody system on a rotating device overnight at 4 ℃. The next day, the agarose beads were washed 3 times and boiled. Samples were conducted by Western blot analysis. The total proteins were separated on SDS-PAGE and transferred onto the PVDF membrane (Merck Millipore, USA). After blocking with 5% milk for 1 h, the PVDF membranes were incubated with primary antibodies overnight at 4 °C. HRP-conjugated GAPDH Monoclonal antibody (Proteintech, HRP-60,004) was used as a loading control.

### Statistical analysis

All data were collected and expressed as the mean ± standard error of the mean of three independent experiments. Tests were analyzed using GraphPad Prism software (version 6, MacKiev Software, Boston, MA, USA), and the comparison between two groups was conducted using a t-test, while the comparison between multiple groups was conducted using one-way analysis of variance (one-way ANOVA). A statistical probability of *P* < 0.05 was considered significant.

### Electronic supplementary material

Below is the link to the electronic supplementary material.

Supplementary Material 1

## Data Availability

Data will be made available on request.
