# Supplementary material for: PGRN is involved in macrophage M2 polarization regulation through TNFR2 in periodontitis
Source: J Transl Med. 2024 Apr 30;22:407. doi: 10.1186/s12967-024-05214-7 (PMC11061905; doi:10.1186/s12967-024-05214-7)
Supplement: Supplementary file 1 — Supplementary Material 1 [file 12967_2024_5214_MOESM1_ESM.docx]

**Supporting Information**

**PGRN is involved in macrophage M2 polarization regulation through TNFR2 in periodontitis**

Liguo Zhang ^1#^, Fujiao Nie ^1#^, Jingjing Zhao ^1^, Shutong Li ^2^, Wenchuan Liu ^1^, Hongmei Guo ^1*^, Pishan Yang ^1*^

^1^ Department of Periodontology & Tissue Engineering and Regeneration, School and Hospital of Stomatology, Cheeloo College of Medicine, Shandong University & Shandong Key Laboratory of Oral Tissue Regeneration & Shandong Engineering Research Center of Dental Materials and Oral Tissue Regeneration & Shandong Provincial Clinical Research Center for Oral Diseases, Jinan, Shandong, 250012, China

^2^ Section of Infection and Immunity, Herman Ostrow School of Dentistry, University of Southern California, Los Angeles, California, USA

^#^ Liguo Zhang and Fujiao Nie contribute equally to this work.

^*^ Corresponding authors: guohm@sdu.edu.cn (H.G.); yangps@sdu.edu.cn (P.Y.)

**Table 1. List of antibodies used for multiplex immunofluorescence**

| Antibody | Source |
| --- | --- |
| CD68 | Abcam (ab201340) |
| CD86 | Cell Signaling Technology (91882) |
| CD206 | Abcam (ab64693) |
| PGRN | Abcam (ab208777; ab187070) |
| TNFR2 | Abcam (ab109322); Proteintech (19272-1-AP) |

**Table 2.** **Primer sequence**

| **Gene** | **Primer sequences（5’-3’）** | **Primer sequences（3’-5’）** |
| --- | --- | --- |
| **GADPH** | GGGTCCCAGCTTAGGTTCAT | CCAATACGGCCAAATCCGTT |
| **IL-6** | CTTCTTGGGACTGATGCTGGT | CTCTGTGAAGTCTCCTCTCCG |
| **iNOS** | GAGACAGGGAAGTCTGAAGCAC | CCAGCAGTAGTTGCTCCTCTTC |
| **TNF-α** | GCCTCCCTCTCATCAGTTCTA | GGCAGCCTTGTCCCTTG |
| **IL-10** | CTGGACAACATACTGCTAACCG | GGGCATCACTTCTACCAGGTAA |
| **Arg-1** | TGTCCCTAATGACAGCTCCTT | GCATCCACCCAAATGACACAT |
| **CD206** | TCAATGCCACTGCCATGCCTAC | AGCTTGCCGTGCGTCTTGC |
| **TNFR2** | ACAAGCGTGCCACGCTGAAGAGG | CTACTCAGTCCTCGCCAATGAGG |


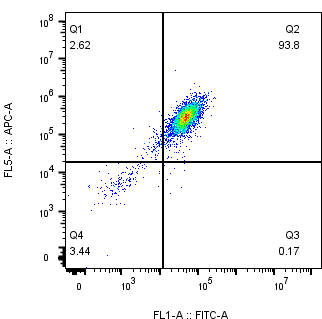


**Figure S1 Identification of BMDMs.**

Double staining result of flow cytometry. The horizontal axis is CD11b, and the vertical axis is F4/80.


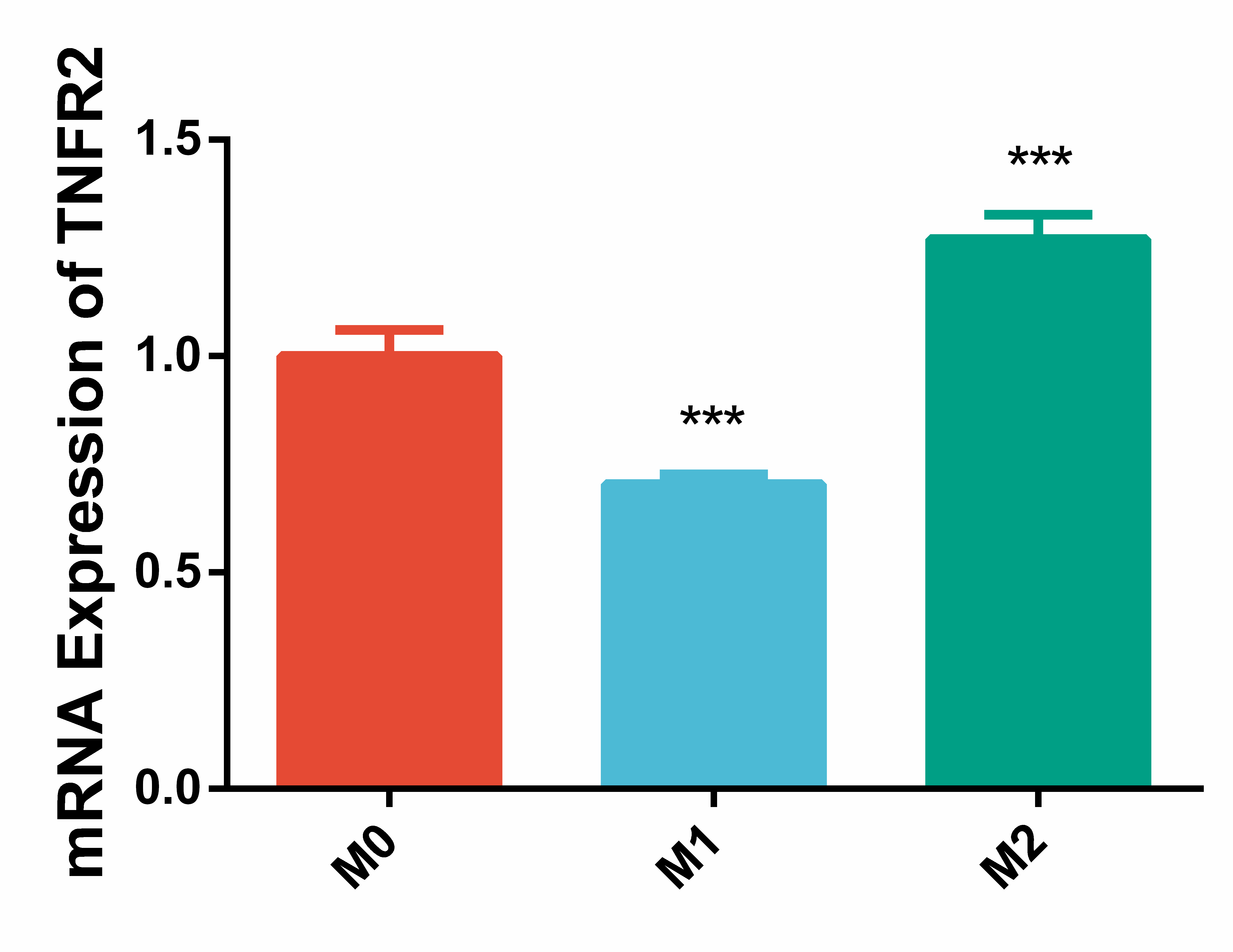


**Figure S2 PCR assay for TNFR2 mRNA expression in M0, M1 and M2**

The mRNA expression of TNFR2 in M0, M1 and M2 (RAW264.7). *** *P* < 0.001
